# The Presence of Cartilage Affects Femoral Rotational Alignment in Total Knee Arthroplasty

**DOI:** 10.3389/fsurg.2022.802631

**Published:** 2022-02-16

**Authors:** Yiming Yang, Xianli Zeng, Yan Jin, Zhemin Zhu, Tsung-Yuan Tsai, Jiarong Chen, Hongyuan Shen, Pingyue Li

**Affiliations:** ^1^Guangdong Key Lab of Orthopedic Technology and Implant, General Hospital of Southern Theater Command of PLA, The First School of Clinical Medicine, Southern Medical University, Guangzhou, China; ^2^Shanghai Key Laboratory of Orthopaedic Implants, Department of Orthopaedic Surgery, Shanghai Ninth People's Hospital, Shanghai Jiao Tong University School of Medicine, Shanghai, China; ^3^School of Biomedical Engineering, Shanghai Jiao Tong University, Shanghai, China; ^4^Engineering Research Center of Clinical Translational Digital Medicine, Ministry of Education of PR China, Shanghai Jiao Tong University, Shanghai, China

**Keywords:** total knee arthroplasty, preoperative planning, cartilage thickness, femoral alignment, posterior condylar angle, mechanical lateral distal femoral angle

## Abstract

**Objective:**

To assess the difference between the posterior condylar angle (PCA) and the mechanical lateral distal femoral angle (mLDFA) in the osseous and cartilaginous contours in a non-arthritic Chinese population.

**Methods:**

Computed tomography (CT) and magnetic resonance imaging (MRI) were obtained from 83 patients with knee injuries before arthroscopy, and femur and distal femoral cartilage three-dimensional (3D) models were constructed. The 3D cartilage model was arranged to share physical space with the 3D femoral model, and then PCA and mLDFA were measured on the osseous and cartilaginous contours, respectively. The differences between the measurements with and without cartilage were evaluated.

**Results:**

The average PCA with cartilage was 2.88 ± 1.35° and without was 2.73 ± 1.34°. The difference was significant in all patients and females but not in males. The average mLDFA with cartilage was 84.73 ± 2.15° and without cartilage was 84.83 ± 2.26°, but the difference was statistically insignificant in all groups.

**Conclusion:**

PCA on the osseous and cartilaginous contours significantly differed with and without cartilage in the female group, suggesting that cartilage thickness should be considered during preoperative femoral rotational resection planning.

## Introduction

Correct component implantation is essential for prosthesis survivorship and clinical outcomes in total knee arthroplasty (TKA). Malalignment jeopardizes patellofemoral and tibiofemoral kinematics and causes instability during knee flexion, imbalanced soft tissues, anterior knee pain, and increased shear force on the patella, which may lead to decreased postoperative satisfaction and revision surgeries (1–4). Several methods have been proposed to perform distal femoral resection, including the surgical transepicondylar axis (sTEA), Whiteside's line, posterior condyle line (PCL) + 3° external rotation for rotational alignment correction, and 5–7° valgus relative to the anatomic axis for improving coronal alignment.

The sTEA is the most approximative flexion axis of the knee, and the symmetric posterior condylar femoral prosthesis implanted parallel to the sTEA is thought to achieve optimal rotational alignment. However, since the sTEA had low intra- and interobserver reproductivity and the anatomic landmarks were difficult to identify during the operation, the PCL + 3° external rotation method was used more often in practice (5). Jang et al. indicated that the PCL + 3° external rotation method was the most accurate compared to the sTEA but highly varied among subjects (6) since the PCA varies remarkably among patients (7).

Regarding mechanical alignment in the TKA surgical technique, distal femoral resection is generally set at a fixed angle from the anatomic axis to make the resection perpendicular to the femoral mechanical axis via intramedullary femoral alignment guides. Many studies documented substantial variations, ranging from 27° varus to 22° valgus, in the distal femoral valgus angle among patients (7, 8), and when the same resected angle was used, postoperative malalignment occurred in 13.8% of cases (8).

Preoperative planning via radiography data, such as X-ray and CT, is advocated by some surgeons to improve postoperative alignment. However, neither X-ray nor CT provides cartilage information. Further, our institute's senior surgeon noticed that patients with mild to moderate arthritis scheduled to undergo TKA had cartilage remnants or intact in the femoral posterior condyles and the unilateral distal femoral condyle, which may result in inaccurate prosthesis implantation. Nam et al. and Asada et al. noted that cartilage remnants influenced Korean and Japanese populations with arthritis (9, 10). However, if cartilage thickness affected the distal femoral resection was unknown, and studies focused on these measurements in the normal Chinese population are lacking. Therefore, our study assessed the effects of cartilage thickness on femoral rotational and coronal alignment in a normal Chinese population. We hypothesized that the presence or absence of cartilage would cause differences in the posterior condylar angle (PCA) and the mechanical lateral distal femoral angle (mLDFA).

## Subjects and Methods

### Radiographic Data

This study recruited 83 patients (mean age 36.2 ± 14.3 years) with a history of sports knee injuries scheduled to undergo primary arthroscopy at our hospital from January 2019 to December 2020. Exclusion criteria included a lower extremity operation history, knee inflammation, lower extremity deformities, and cartilage wear on the femoral posterior and distal condyles found during knee arthroscopy.

This retrospective study was approved by the institutional review committee of People's Liberation Army General Hospital of Southern Theater Command, and all the subjects recruited signed a medical informed consent document preoperatively and underwent lower extremity CT scanning (SOMATOM Emotion 16, Siemens Healthcare Gmbh, Germany; slice thickness = 1 mm) and a magnetic resonance imaging (MRI) scan of the injured knee (GE 3.0T, USA; series of O Sag 3D-FS-SPGR, matrix = 256 × 256, flip angle = 15°, slice thickness = 1 mm). The radiography images were exported as Digital Imaging and Communications in Medicine (i.e., DICOM) files and imported into Amira 6.7 (Thermo Fisher Scientific, Rockford, IL, USA) to construct a three-dimensional (3D) femoral model and the homolateral femoral cartilage 3D model. Subsequently, both were imported into the MATLAB (Mathworks Inc., Natick, MA, USA), and the cartilage 3D model was moved as the float to share physical space with the femoral 3D model (the fixed object) using the best fit method.

### Coordinate System

**Figure 1:**
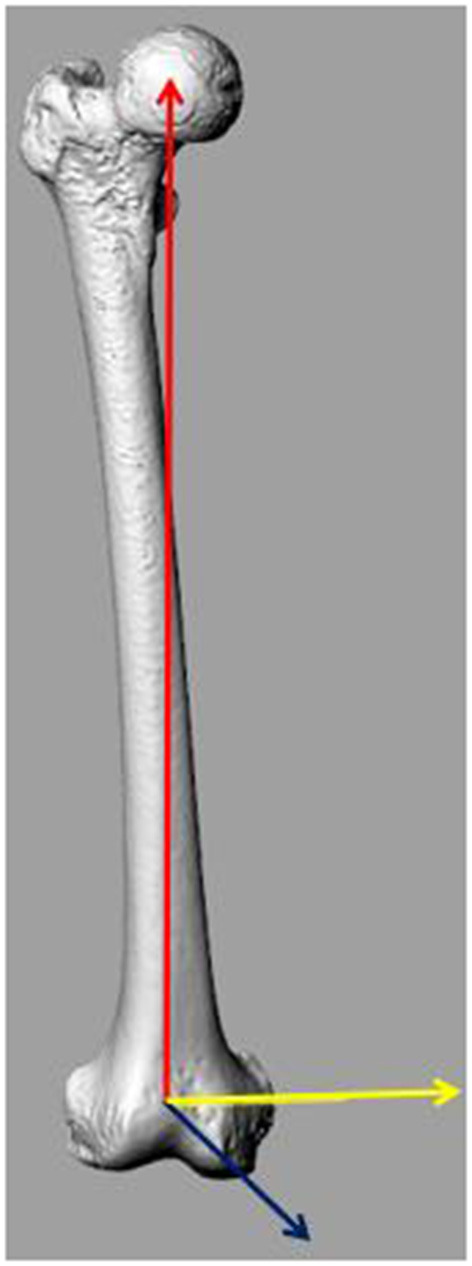
Femur coordination. The mechanical axis (MA) is defined as the line connecting the geometric center of the femoral head and the midpoint of the surgical transepicondylar axis (sTEA) (i.e., Y-axis; the red line). The sTEA is indicated by the yellow line. The Z-axis plane is created perpendicular to the MA, passing the midpoint of the sTEA. The X-axis (the blue line) is perpendicular to the plane formed by the Y- and Z-axes.

The femoral 3D models were imported into Rhinoceros software 5.0 (Robert McNeel & Associates, USA). The sTEA was defined as the line connecting the most prominent point of the lateral epicondyle and the sulcus point of the medial epicondyle. The femoral mechanical axis (MA) was defined as the line connecting the geometric center of the femoral head to the midpoint of the sTEA. The plane was then created perpendicular to the MA, passing through the midpoint of the sTEA. The MA was regarded as the Y-axis and the TEA as the Z-axis, and the X-axis was perpendicular to the plane formed by the Y- and Z-axes (Figure 1).

### Measurements

**Figure 2:**
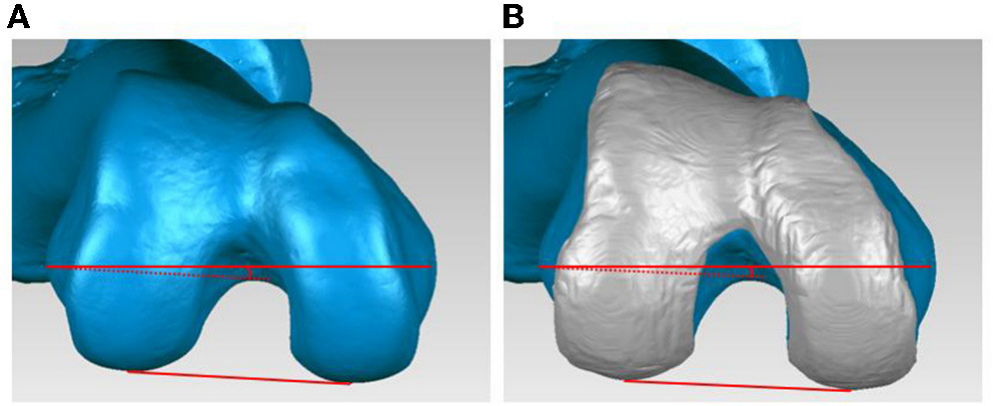
The posterior condylar angle (PCA). The PCA was defined as the angle between the surgical transepicondylar axis and the posterior condyle line, which connects the most posterior points of the osseous and the cartilaginous contours, respectively, of the lateral and medial posterior condyles on the transverse plane perpendicular to the Y-axis. **(A)** PCA on the osseous contours. **(B)** PCA on the cartilaginous contours.

**Figure 3:**
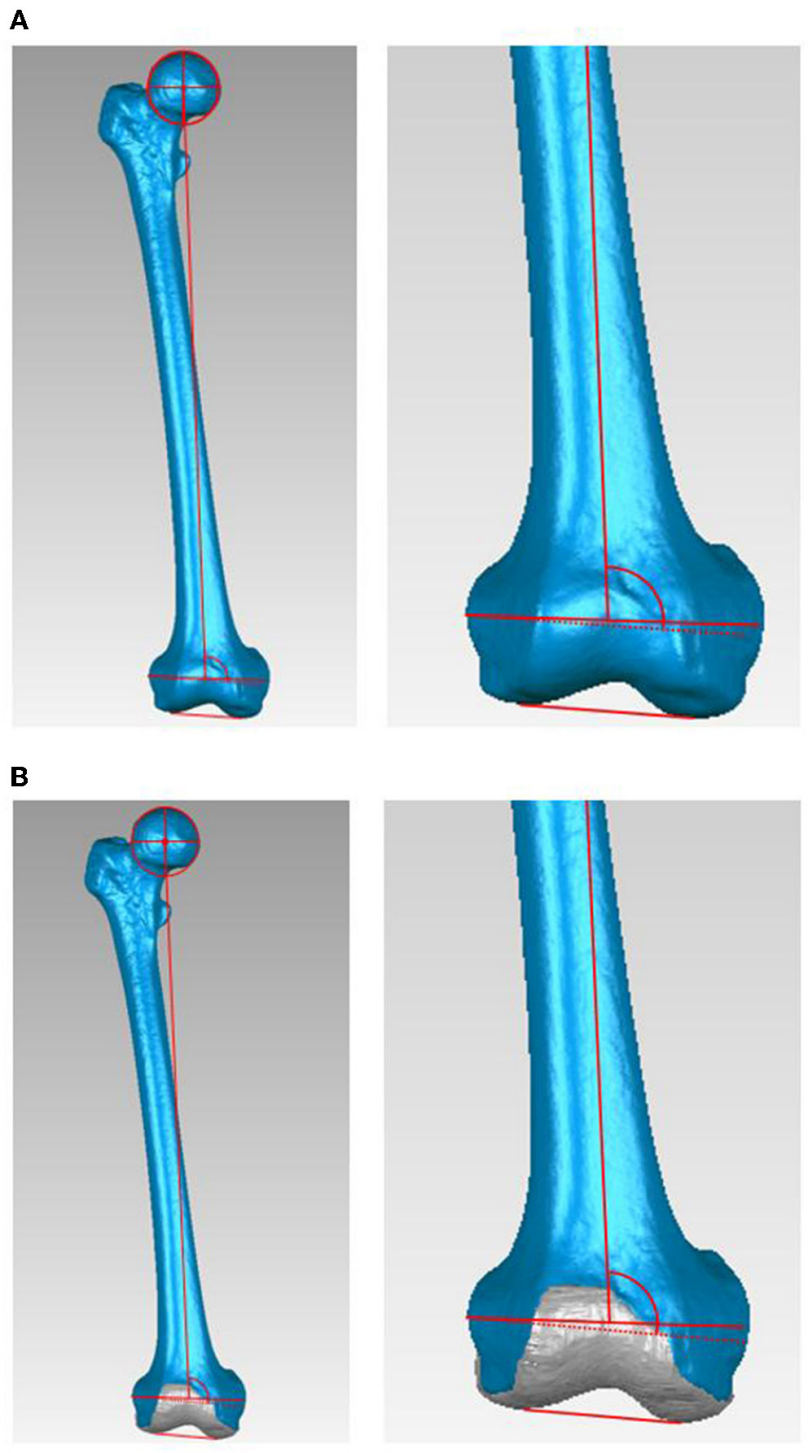
The mechanical lateral distal femoral angle (mLDFA). The mLDFA was defined as the angle between the femoral mechanical axis and the line connecting the most distal point of the osseous and the cartilaginous contours, respectively, of the lateral and medial distal condyles on the coronal plane perpendicular to the Z-axis. **(A)** The mLDFA on the osseous contours. **(B)** The mLDFA on the cartilaginous contours.

The PCA was defined as the angle between the sTEA and the PCL, which connects the most posterior points of the osseous and cartilaginous contours of the lateral and medial posterior condyles on the transverse plane perpendicular to the Y-axis (Figure 2). The mLDFA was defined as the angle between the femoral MA and the line connecting the most distal point of the osseous and the cartilaginous contours, respectively, of the lateral and medial distal condyles on the coronal plane perpendicular to the Z-axis (Figure 3). The patient was considered an outlier if the PCA deviated more than 2° from the average PCA measurement or the mLDFA deviated more than 3° from the average mLDFA measurement, as alignment ranging from 3° varus to 3° valgus from the mechanical axis and 2° external to 2°internal rotation from the PCA are desired and associated with better clinical outcomes (7, 8).

The cartilage thickness on the posterior femoral condyle was measured at the distal femoral resection plane, which was parallel to the coordinate axial plane, passing the most posterior points defining the PCL. The cartilage thickness on the distal femoral condyle was measured at the coordinate coronal plane passing the most distal points.

Three months after the initial examination, ten subjects (five males and five females) were measured again by the same observer and a different observer to assess the inter- and intra-observer differences. The intra-observer significance was 0.89, and the inter-observer significance was 0.86 using the intra-class correlation method.

### Statistical Analyses

Statistical analyses were performed using SPSS software (version 22.0, IBM Corp. Armonk, NY, USA) and G-power software (version 3.1, Düsseldorf, Germany). All measurements were reported as the means ± standard deviations. An independent *t*-test was performed to determine the differences in cartilage thickness between the lateral and medial sides, and the paired *t*-test was used to determine the differences between the PCA and mLDFA with and without cartilage. A *post-hoc* power analysis (rather than a *pre-hoc*) was used to determine if the number of subjects was sufficient to detect statistically significant differences between the two groups. A *p*-value of < 0.05, an effect size of 80%, and a 5% type I error risk were considered statistically significant.

## Results

The average PCA in all patients with cartilage was significantly larger than without cartilage (with: 2.88 ± 1.35°, range, 0.07 to 6.01°; without: 2.73 ± 1.34°, range, −0.11 to 6.05°; Table 1). There was a similar result in the female group but not in the male group. PCA outliers accounted for 11 % of the population with cartilage and 12 % without cartilage.

**Table 1:** The posterior condylar angle (PCA) and the mechanical lateral distal femoral angle (mLDFA).

**Cartilage**	**PCA**			**mLDFA**		
	**Total**	**Male**	**Female**	**Total**	**Male**	**Female**
With (°)	2.88 ± 1.35	2.83 ± 1.07	2.93 ± 1.63	84.73 ± 2.15	85.42 ± 1.76	83.91 ± 2.30
Without (°)	2.73 ± 1.34	2.73 ± 1.14	2.72 ± 1.56	84.83 ± 2.26	85.59 ± 1.82	83.92 ± 2.42
*P*-value	0.020	0.237	0.041	0.128	0.052	0.875

The average mLDFA with cartilage was 84.73 ± 2.15° (range, 78.60 to 90.33°) and without cartilage was 84.83 ± 2.26° (range, 78.48 to 90.78°), but the difference between the groups was statistically insignificant. The results were similar in the male and female groups (Table 1). mLDFA outliers accounted for 13 % of the population with cartilage and 17 % without cartilage.

The cartilage thickness between the lateral and medial sides of the distal and posterior condyles did not differ in all patients or the male and female groups (Table 2).

**Table 2:** Cartilage thickness (mm).

**Cartilage thickness**	**Posterior femoral condyle**			**Distal femoral condyle**		
	**Total**	**Male**	**Female**	**Total**	**Male**	**Female**
Medial	1.98 ± 0.49	2.14 ± 0.49	1.79 ± 0.42	1.87 ± 0.46	2.06 ± 0.40	1.64 ± 0.42
Lateral	2.09 ± 0.47	2.29 ± 0.46	1.86 ± 0.39	1.85 ± 0.44	1.98 ± 0.39	1.69 ± 0.45
*P*-value	0.138	0.159	0.417	0.830	0.361	0.572

The *post-hoc* power analysis indicated that the study size was sufficient to detect significant differences in all measurements, and the group size to achieve sufficient power (>0.80) was acceptable.

## Discussion

In this study, PCA was significantly larger with cartilage than without cartilage for all patients and in females but not in males. There was also no difference in the mLDFA between the osseous and cartilaginous contours. To our knowledge, this is the first study to assess the effect of cartilage thickness on femoral mechanical and rotational alignments. Not considering the cartilage thickness may lead to femoral malrotation implantation in females.

Various techniques have been used to perform proper TKA alignment, including matching the component with human natural anatomic-like, gender-specific, and patient-specific prosthesis, intraoperative computer navigation, and robot assistance (11). Regardless, preoperative planning plays a crucial role in achieving proper alignment. Full-length plain films of both lower limbs and CT slices were first used to visualize various knee joint parameters, but these measurements were dependent on two-dimensional (2D) models. However, Okamoto et al. found that 2D preoperative planning resulted in the internal rotation of the femoral component, and the clinical and surgical angle values measured based on X-ray and CT slices would be smaller than the 3D measurements, causing internal component rotation (12). Numerous studies have been conducted to measure relative TKA parameters from 3D models based on CT and MRI, all of which confirmed the high inter-subject variation of PCA (7, 8, 13). Gokhan et al. indicated that the average PCA was 3.3 ± 1.5° via 3D constructed models based on CT scans, with 2.8 % of scans identified as outliers. That PCA average is larger than ours, possibly owing to different ethnicities. A smaller population could have also led to a higher proportion of outliers (8). However, conventional CT cannot reveal the knee joint cartilage, resulting in missing information on cartilage thickness. Asada et al. reported osteoarthritic femur measurements in the axial plane of CT images after injecting medication into the knee, allowing for cartilage visualization. The average PCA was 2.2° with cartilage and 3.3° without (10), but their results were based on 2D radiography. Koh et al. indicated that the PCA was 2.2 ± 1.0° internally rotated relative to the sTEA through 3D bony and cartilaginous models based on MRI scans of end-stage osteoarthritis patients (13). Nam et al. also reported that the PCA was 2.4 ± 0.9° with cartilage and 2.6 ± 1.0° without via femoral 3D MRI constructed models, and the variation was more evident in females, similar to our results (9). When performing femoral rotational resection, the same cutting angle leads to over 10% outliers, possibly resulting in malalignment. Even though our results indicated that the proportion of outliers would be lower if cartilage was considered, this trend requires confirmation in further studies.

For coronal alignment of the femur, long-leg radiographs were obtained to measure the angle between the mechanical axis and anatomical axis, mLDFA, and anatomical LDFA. Maderbacher et al. found that the radiograph parameters changed when the leg rotational positions were altered (14). Degen et al. reported that the average mLDFA was 87.2 ± 2.1° in 3D lower limb bone models of a Caucasian population based on CT data (15). To our knowledge, this is the first study to explore the relationship between cartilage thickness and mLDFA. Our subjects had relatively normal knee joints. Therefore, despite a statistically insignificant difference between the measurements with and without cartilage, the effect of cartilage thickness on mLDFA in the osteoarthritis cases, especially with cartilage wear in the medial or lateral compartments, requires further study.

In conventional mechanical alignment, the TKA components are implanted perpendicular to the mechanical axis of the lower extremity to achieve a neutral prosthetic joint mechanical alignment. The distal resection angle is set based on the angle between the femoral mechanical axis and the anatomical axis, and the posterior cutting angle is set according to the PCA when using the PCL + 3° external rotation as the reference line. Our results indicated that cartilage thickness might influence the femoral rotation, perhaps causing component malalignment.

In kinematic alignment, after compensating for cartilage wear, symmetric resections of the femoral distal condyles are performed, and the transverse rotation angle is set in line with PCA (16). In classic mechanical alignment, the distal femoral resection is performed perpendicular to the mechanical axis, in which the natural joint line changes. However, recent studies proposed that kinematical prosthesis alignment obtains more physiological tibial internal rotation by restoring the natural mLDFA (17). To our knowledge, few studies have focused on the mLDFA in a normal Chinese population on 3D models. Our study presented the average mLDFA in a normal Chinese population and indicated that the cartilage thickness does not considerably affect the mLDFA. Thus, MRI might not be necessary for preoperative planning of the coronal alignment. However, as these data were extracted from non-arthritic femurs, differences might exist in single-compartment osteoarthritic femurs.

We also evaluated the cartilage thickness in the distal and posterior femoral condyles and found no difference between the lateral and medial sides. Nam et al. reported that the lateral posterior cartilage thickness (2.2 ± 1.0 mm) was significantly greater than the medial side (1.8 ± 0.4 mm), and Asada et al. reported 1.5 ± 0.39 mm (9, 10). However, both studies enrolled arthritic subjects where pathological cartilage wear or defects were expected. A similar trend was reported by Si et al., who reported 1.75 ± 0.12 mm for the medial distal femoral cartilage and 1.77 ± 0.13 mm for the lateral, and 1.59 ± 0.16 mm for the medial posterior femoral cartilage and 1.61 ± 0.22 mm for the lateral (18). The thickness difference could be explained by the subjects we recruited, who were involved in high-intensity physical activities (19). This measurement provides information for kinematic alignment TKA to compensate for cartilage wear.

Our study had several limitations. First, the number of subjects was small, so the average PCA and mLDFA might not represent the whole Chinese population. Further, ethnicity restricts these data from extending to Caucasian populations, so data expansion is necessary. Secondly, our subjects were not completely “normal” since they all had a history of knee injuries, which may have affected the data. However, we confirmed that there was no cartilage wear via arthroscopy. Finally, the thickness of the constructed cartilage could not be determined by measuring the actual cartilage thickness.

## Conclusion

The PCA of the osseous and cartilaginous contours in females significantly differed, suggesting that cartilage thickness should be considered during preoperative TKA planning to restore the natural distal femoral rotation. Additionally, cartilage thickness did not influence the mLDFA. However, how cartilage thickness affects the mLDFA in osteoarthritic patients requires further exploration, especially in patients with single-compartment osteoarthritis. The rotational and mechanical alignment angle was fixed during TKA. Thus, malalignment would occur in some patients regardless. The resection should be individualized and decided on during patient-specific preoperative planning to obtain the optimal femoral implant alignment.

## Data Availability Statement

The raw data supporting the conclusions of this article will be made available by the authors, without undue reservation.

## Ethics Statement

The studies involving human participants were reviewed and approved by People's Liberation Army General Hospital of Southern Theatre Command. The patients/participants provided their written informed consent to participate in this study. Written informed consent was obtained from the individual(s) for the publication of any potentially identifiable images or data included in this article.

## Author Contributions

The first draft of the manuscript was written by YY. Data collection and analysis were performed by XZ. Interpretation of data was performed by YJ, ZZ, HS, PL, JC, and T-YT had substantively revised it. All authors contributed to the study, collected the data, read, and approved the final manuscript

## Funding

This work was supported by the National Natural Science Foundation of China [Grant Number: 81871808].

## Conflict of Interest

The authors declare that the research was conducted in the absence of any commercial or financial relationships that could be construed as a potential conflict of interest.

## Publisher's Note

All claims expressed in this article are solely those of the authors and do not necessarily represent those of their affiliated organizations, or those of the publisher, the editors and the reviewers. Any product that may be evaluated in this article, or claim that may be made by its manufacturer, is not guaranteed or endorsed by the publisher.
